# Comparison of Demographic, Clinical, and Echocardiographic Features Between Complete and Incomplete, and Early and Late Presenters of Kawasaki Disease: A 10-Year Single-Center Experience

**DOI:** 10.7759/cureus.45819

**Published:** 2023-09-23

**Authors:** Naser Kolko, Yasser A Bhat, Abdulrahman Al Mesned, Abdullah Al Qwaee, Ali Al Akhfash, Marwan Alhobani, Ibrahim Al Anazi

**Affiliations:** 1 Pediatric Cardiology, Prince Sultan Cardiac Center, Buraidah, SAU; 2 Cardiac Intensive Care, Prince Sultan Cardiac Center, Buraidah, SAU; 3 General Pediatrics, Maternity and Children Hospital, Buraidah, SAU

**Keywords:** intravenous immunoglobulin, coronary artery aneurysm, late presenters, early presenters, incomplete kawasaki, complete kawasaki, kawasaki disease

## Abstract

Introduction: The diagnosis of Kawasaki disease (KD) is based mainly on clinical findings and supported by laboratory tests. Complete KD fulfills the main clinical criteria, while incomplete KD includes patients with fewer main criteria and compatible laboratory or echocardiographic findings. The study compares the demographic, clinical, laboratory, and echocardiographic parameters between the complete and incomplete KD and early and late presenters. Moreover, it describes the coronary manifestations of the study population.

Methodology: A retrospective review of all patients admitted with a diagnosis of KD during the period from January 2010 to September 2020 was conducted. Clinical presentation, laboratory features, echocardiographic observations, and follow-up data were examined. Moreover, the patients were further classified as early presenters (presented within 10 days of fever onset) and late presenters (presented after 10 days of disease onset). A comparison between complete and incomplete KD and early and late presenters was performed for demographic, clinical, and echocardiographic features.

Results: A total of 76 patients were admitted with a diagnosis of KD. The median age of presentation was 28 months, with a range of five to 144 months, and the median timing was seven days, with a range of one to 30 days. The median follow-up period was six weeks, with a range of one to 192 weeks. Complete KD was present in 38 patients (50%), and 38 (50%) had incomplete KD. Skin manifestations, oral mucosal changes, skin desquamation, conjunctivitis, and lymphadenopathy were present more in patients with complete KD than incomplete ones. Complete and incomplete diseases did not differ regarding coronary artery lesions. Of the patients, 53 (70%) presented 10 days or less after the onset of fever, and 23 (30%) presented after the 10th day of disease onset. Comparison between early and late presenters revealed significantly greater mucus membrane changes and lymphadenopathy manifestations among the early presenters and coronary artery lesions among the late presenters.

Conclusion: The clinical features of KD should prompt early referral for evaluation, echocardiography, and early administration of intravenous immunoglobulin to prevent coronary artery complications. The complete form of Kawasaki does not have more frequent coronary artery lesions than the incomplete form. Additionally, late presenters may be at increased risk for coronary artery abnormalities than early presenters.

## Introduction

The diagnosis of Kawasaki disease (KD) is based mainly on clinical findings and supported by laboratory tests to avoid misdiagnosis with other syndromes with similar presentation [1-3]. Complete KD fulfills the main clinical criteria of having a fever that lasts for at least five days, with at least four of the five principal clinical features: (1) lip erythema and cracking, strawberry tongue, and/or oral and pharyngeal mucosa erythema; (2) bilateral bulbar conjunctival injection with no exudate; (3) maculopapular rash, diffuse erythroderma, or erythema multiforme-like rash; (4) erythema and edema of the hands and feet in the acute phase and/or periungual desquamation in the subacute phase; (5) cervical lymphadenopathy (≥1.5 cm diameter), usually unilateral. Incomplete KD includes patients with fewer main criteria and compatible laboratory or echocardiographic findings. KD is unlikely if acute phase reactants like erythrocyte sedimentation rate (ESR), C-reactive protein (CRP), and platelet count are within normal limits after seven days of illness [4,5].

The dreadful complication of KD is a coronary artery aneurysm (CAA), which occurs in 15-25% of patients without intravenous immunoglobulin (IVIg) treatment. The incidence of CAA is reduced to 5% if IVIg is given within 10 days of disease onset [6]. A second dose of immunoglobulin and corticosteroids is administered in patients who are refractory to the initial treatment. At the same time, adjunctive drugs, such as infliximab, are seen as a factor in reducing the duration of fever but may not improve treatment outcomes [7-9]. Managing cardiovascular complications of KD is challenging in pediatric cases, particularly when there are coronary artery aneurysms and coronary artery obstruction [10,11].

The study compares the demographic, clinical, laboratory, and echocardiographic parameters between the complete and incomplete KD and early and late presenters. Moreover, it describes the coronary outcomes of the study population.

## Materials and methods

A retrospective study was conducted in the general pediatrics and pediatric cardiology departments at Maternity and Children Hospital and Prince Sultan Cardiac Centre, Qassim, respectively. The institutional review board approved the study (IRB Approval No.: 21-1006; date of approval: January 4, 2021). The medical records of all patients with KD manifestations between January 2010 and September 2020 were retrieved and retrospectively analyzed. Patients admitted with clinical features suggesting KD were included depending on the diagnostic guidelines by the American Heart Association for KD [5]. Diagnosis of complete KD was based on the presence of persistent fever that lasted for a period of a minimum of five days along with four of the main signs and symptoms in the following list: noticed changes in extremities such as erythema in hands or soles, sometimes accompanied by skin induration that may occur in the acute phase; desquamation of fingertips and toes occurring within two to three weeks after fever onset; polymorphous exanthem; bilateral bulbar conjunctival injection without exudate; changes in lips and oral cavity, including erythema and lips cracking, strawberry tongue, diffuse injection of oral and pharyngeal mucosa; cervical lymphadenopathy (≥1.5 cm in diameter) usually manifested unilaterally. Incomplete KD was identified if the fever lasted for a minimum of five days, with at least two of the main criteria in conjunction with laboratory findings indicating the presence of severe systemic inflammation [4].

All patients with other proven diagnoses were excluded. The patient data were reviewed from the medical records' old file archive, electronic health information system, pediatric cardiology database, and pediatric echocardiography records. Data collected included demographic data, age and date of presentation, clinical features on presentation, laboratory markers, presentation timing after the onset of fever, intravenous administration of IVIg and timing of administration, doses of IVIg administered, echocardiographic findings, and coronary artery involvement. Patients were divided into two groups (complete and incomplete KD) based on the clinical findings and the fulfillment of the diagnostic criteria [4,5]. Patients fulfilling the criteria were labeled as having complete KD, while patients with less than four major criteria were labeled incomplete. Patients were further divided into early and late presenters based on the presentation timing after the onset of the fever (early if presented within 10 days of onset of fever, and late if presented after the 10th day of onset of disease). A comparison between complete and incomplete KD and early and late presenters was performed, focusing mainly on the demographic, clinical, and echocardiographic findings.

Data analysis

IBM SPSS Statistics 26 software (IBM Corp., Armonk, NY) was used for statistical analysis. Numerical variables were expressed as median, categorical variables as numbers, and percentages as appropriate. The independent-sample t-test was used to compare numerical variables, and the Qi-square test to compare categorical variables.

## Results

During the study period, 76 patients were admitted with a diagnosis of KD. The male-to-female ratio was 2.5 to 1 (54 males and 22 females). Complete KD was present in 38 patients (50%), and 38 had incomplete KD. Tables 1, 2 show the study group's demographic, clinical, and laboratory features.

**Table 1:** Demographic, echocardiographic, and clinical features of the study group (categorical variables)

Characteristics		N	%
Gender	Male	54	71
	Female	22	28.9
Coronaries	Normal coronaries	56	73.6
	Abnormal coronaries	20	26.3
Temperature (Celsius)	<37	8	10.5
	>37	68	89.4
Skin manifestations	Yes	60	78.9
	No	16	21
Skin desquamation	Yes	33	43.4
	No	43	56.5
Lymphadenopathy	Yes	50	65.7
	No	26	34.2
Conjunctivitis	Yes	54	71
	No	22	28.9
Oral mucosa changes	Yes	63	82.8
	No	13	17.1

**Table 2:** Demographic and clinical characteristics of the patient population (continuous variables; N = 76)

		Age (months)	Timing of presentation after fever (days)	Follow-up period (weeks)
	Valid (N)	75	76	54
	Missing (N)	1	0	22
Median		28	7	6
Range		5-144	1-30	1-192
Standard deviation		35.19	6.64	33.10

Skin manifestations, oral mucosal changes, skin desquamation, conjunctivitis, and lymphadenopathy were present more in patients with complete KD than incomplete ones. In contrast, the two groups showed no difference in age, presentation timing, and laboratory markers. Furthermore, patients with complete and incomplete diseases did not differ regarding coronary artery lesions (Tables 3, 4).

**Table 3:** Comparison of demographic, clinical, echocardiographic, and treatment features between complete and incomplete Kawasaki disease patients # P-value (significant) > 0.05.

Characteristics		Complete, N = 38, (50%)	Incomplete, N = 38, (50%)	P-value^#^
Gender	Male	29 (76.3)	25 (65.7)	0.448
	Female	9 (23.6)	13 (34.2)	
Echocardiography	Normal coronaries	26 (68.4)	30 (78.9)	0.435
	Abnormal coronaries	12 (31.5)	8 (21)	
Temperature (Celsius)	<37	2 (5.2)	6 (15.7)	0.262
	>37	36 (94.7)	32 (84.2)	
Skin manifestations	Yes	35 (92.1)	25 (65.7)	0.01
	No	3 (7.8)	13 (34.2)	
Oral mucosa changes	Yes	35 (92.1)	26 (68.4)	0.02
	No	3 (7.8)	12 (31.5)	
Skin desquamation	Yes	24 (63.1)	9 (23.6)	0.001
	No	14 (36.8)	29 (76.3)	
Lymphadenopathy	Yes	35 (92.1)	15 (39.4)	0.001
	No	3 (7.8)	23 (600.5)	
Conjunctivitis	Yes	36 (94.7)	18 (47.3)	0.001
	No	2 (5.2)	20 (52.6)	

**Table 4:** Comparison of demographics and clinical and laboratory features between complete and incomplete Kawasaki disease (N = 76) µL, microliter; g/dL, grams per deciliter; mm/hr, millimeters per hour; mg/dL, milligrams per deciliter. † Data are not available for some patients.

Characteristic		N (%)	Mean	STD	P-value
^†^Age (months)	Complete	38 (50)	36.87	29.19	0.306
	Incomplete	37 (48.6)	45.24	40.41	
Timing of presentation after fever (days)	Complete	38 (50)	7.68	5.54	0.223
	Incomplete	38 (50)	9.58	7.56	
^†^Total white cell count (× 10^3 ^/µL)	Complete	37 (48.6)	11.70	9.04	0.816
	Incomplete	38 (50)	12.16	8.15	
^†^Hemoglobin (g/dL)	Complete	37 (48.6)	14.94	10.22	0.353
	Incomplete	38 (50)	12.99	7.65	
^†^Platelets (× 10^3^/µL)	Complete	35 (46)	430.83	192.59	0.291
	Incomplete	37 (48.6)	497.89	322.28	
^†^Erythrocyte sedimentation rate (mm/hr)	Complete	37 (48.6)	74.43	36.55	0.296
	Incomplete	37 (48.6)	64.65	43.06	
^†^C-reactive protein (mg/dL)	Complete	33 (43.4)	220.70	258.74	0.294
	Incomplete	35 (46)	159.59	216.82	
^†^Albumin (g/dL)	Complete	33 (43.4)	50.64	35.65	0.918
	Incomplete	36 (47.3)	51.52	35.35	

Of the patients, 59 (77.6%) presented 10 days or less after the onset of fever, and 17 (22.3%) presented after the 10th day of disease onset. Comparison between early and late presenters revealed significantly greater mucus membrane changes and lymphadenopathy manifestations among the early presenters and coronary artery lesions among the late presenters (Table 5).

**Table 5:** Comparison between early and late presenters for demographic, clinical, and echocardiographic variables

Variables		Early presenters, N = 59 (77.6%)	Late presenters, N = 17 (22.3%)	P-value
Gender	Male	42 (73.6)	12 (70.5)	1.00
	Female	17 (28.8)	5 (29.4)	
Temperature (Celsius)	<37	5 (8.4)	3 (17.6)	0.367
	>37	54 (91.5)	14 (82.3)	
Skin manifestations	Yes	47 (79.6)	13 (76.4)	0.764
	No	12 (20.3)	4 (23.5)	
Skin desquamation	Yes	26 (44.1)	7 (41.1)	1.00
	No	33 (55.9)	10 (58.9)	
Lymphadenopathy	Yes	43 (72.8)	7 (41.1)	0.021
	No	16 (27.1)	10 (58.9)	
Conjunctivitis	Yes	45 (76.2)	9 (52.9)	0.075
	No	14 (23.7)	8 (47.1)	
Diagnosis	Complete	32 (54.2)	6 (35.3)	0.271
	Incomplete	27 (45.7)	11 (64.7)	
Coronary artery involvement	Normal coronaries	47 (79.6)	9 (52.9)	0.057
	Abnormal coronaries	12 (20.3)	8 (47.1)	

A comparison of demographic, clinical, and laboratory abnormalities between the affected coronary artery and normal coronary artery groups did not reveal any differences (Tables 6, 7).

**Table 6:** Comparison between patients with normal and abnormal coronary arteries for demographic and clinical variables P-value (significant) < 0.05.

		Normal coronary artery findings, N = 56 (73.6%)	Abnormal coronary artery findings, N = 20 (26.3%)	P-value
Gender	Male	40 (71.4)	14 (70)	0.767
	Female	16 (28.5)	6 (30)	
Temperature (Celsius)	<37	4 (7.1)	4 (20)	0.672
	>37	52 (92.8)	16 (80)	
Skin manifestations	Yes	45 (80.3)	15 (75)	1.00
	No	11 (19.6)	5 (15)	
Skin desquamation	Yes	21 (37.5)	12 (60)	0.097
	No	35 (62.5)	8 (40)	
Conjunctivitis	Yes	40 (71.4)	14 (70)	0.767
	No	16 (28.5)	6 (30)	
Lymphadenopathy	Yes	40 (71.4)	10 (50)	0.15
	No	16 (28.5)	10 (50)	
Complete	Complete	26 (46.4)	12 (60)	0.399
	Incomplete	30 (53.5)	8 (40)	

**Table 7:** Comparison between patients with normal and abnormal coronary arteries for demographic and laboratory parameters † Data are not available for some patients.

Variables (mean)	Normal coronary artery findings, N = 56 (73.6%)	Abnormal coronary artery lesions, N = 20 (26.3%)	P-value
^†^Age (months)	44.51	31.35	0.153
Timing of presentation after fever (days)	7.62	11.25	0.037
^†^Total white cell count (× 10^3 ^/µL)	11.45	13.36	0.402
^†^Hemoglobin (g/dL)	14.64	11.92	0.257
^†^Platelets ( × 10^3 ^/µL)	438.57	545.44	0.143
^†^Erythrocyte sedimentation rate (mm/hr)	67.19	76.35	0.393
^†^C-reactive protein (mg/dL)	182.68	208.96	0.697
^†^Albumin (g/dL)	51.46	50.05	0.885

The coronary arterial abnormalities were described as prominent, dilatation, ectasia, and aneurysm. The absolute coronary artery dimensions ranged from 2 to 5 mm. Among the patients with coronary affection, follow-up was available for nine (41%) patients, and all showed regression of coronary artery abnormalities. The echocardiographic abnormalities of patients with coronary artery affection are shown in Table 8.

**Table 8:** Echocardiographic findings in the patients having coronary artery affection (N = 20) LAD, left descending coronary artery; LMC, left main coronary artery; RCA, right coronary artery.

Variables		N	%
Coronary artery abnormality (dilatation, ectasia, aneurysm)	LAD	6	30
	LMC	4	20
	LAD, LMC	3	15
	LAD, RCA	3	15
Aortic root dilatation		2	10
Mitral regurgitation		1	5
Pericardial effusion		1	5
Depressed cardiac function		1	5

## Discussion

KD is a medium vessel vasculitis with a predilection for coronary arteries and has been recognized to be the most common cause of acquired heart disease in children. KD is now being increasingly recognized in several developing countries. Even though more than 50 years have passed since the first case of KD was identified by Dr. Tomisaku Kawasaki, the diagnosis of KD remains a clinical dilemma, and there is no confirmatory laboratory test [11]. In the current study, fever (89.47%) and mucous membrane changes (82.89%) were the commonest manifestations, followed by skin manifestations (78.95%), while cervical lymphadenopathy was the least common sign (65.79%). However, a study by Wang et al. reported mucus membrane changes and lymphadenopathy as the most common clinical features [12]. Comparing clinical parameters between complete and incomplete forms revealed a higher occurrence of oral mucosa changes, skin manifestations, conjunctivitis, and lymphadenopathy among the complete Kawasaki patients. Further comparison between the two groups revealed no difference in laboratory parameters like total white cell count, platelet count, CRP, erythrocyte sedimentation rate, and albumin. Maric et al. [13] recorded a higher frequency of skin and mucous membrane changes as well as higher serum bilirubin, aminotransferases, gamma-glutamyl transferase, and lactate dehydrogenase levels in the complete Kawasaki group. Male gender may have an increased risk of developing coronary artery aneurysms [14]; however, no significant difference was found between males and females regarding coronary artery abnormalities in the current study. None of the laboratory parameters suggested an increased risk of coronary artery affection. All laboratory parameters revealed no significant difference when compared between patients with no coronary artery abnormalities and those with them. In contrast, Yu-Mi Seo et al. [15] compared the laboratory values in 615 Kawasaki patients. They found that patients with coronary artery lesions had higher CRP and neutrophil differential counts but lower hemoglobin and albumin levels than those without coronary artery abnormalities. Similarly, Jeon et al. [16] noticed lower hemoglobin and albumin levels and higher CRP and platelets in the coronary artery lesion group. Late presenters had more frequent coronary artery lesions in this study than early presenters. Other authors documented similar findings [16,17]. This study shows a high incidence of coronary artery abnormalities, around 26.6%, after IVIg use. Out of 17 patients who presented late, eight patients (47%) had abnormal coronaries, and among the early presenters (N = 59), 12 patients (20%) had coronary artery lesions. Dominguez et al. [18] observed coronary artery abnormalities in 27% of the patients despite IVIg use. The authors stressed early diagnosis and treatment to impact the high incidence of coronary artery lesions.

The study analyzed the clinical signs, laboratory features, and coronary abnormalities associated with KD in the Qassim region of Saudi Arabia. The study was done in a tertiary hospital in one of the regions of Saudi Arabia, and a nationwide study is necessary to investigate the overall clinical-laboratory profile and coronary outcomes of KD in Saudi Arabia.

Limitations

The study shows that 27% of the patients had coronary artery affection even after IVIg use, which does not align with current evidence of coronary artery abnormalities in less than 5%. The higher incidence could have been due to sampling bias; therefore, this sample could underestimate patients with normal coronary arteries. The advanced echocardiography devices and better-quality images could also cause false positive detection of coronary artery dilatation and ectasia.

## Conclusions

The clinical features of KD should prompt early referral for evaluation, echocardiography, and early administration of IVIg to prevent coronary artery complications. The complete form of Kawasaki does not have more frequent coronary artery lesions than the incomplete form. Moreover, early presenters have more favorable coronary manifestations than late presenters. Delays in the diagnosis and administration of IVIg can lead to significant coronary artery abnormalities and potential morbidity.

## References

[1] Lowry A, Myones B, Tran J, Han Y (2008). Guidelines for echocardiography of low-risk patients with Kawasaki disease. Pediatrics.

[2] Marchesi A, Tarissi de Jacobis I, Rigante D (2018). Kawasaki disease: guidelines of the Italian Society of Pediatrics, part I - definition, epidemiology, etiopathogenesis, clinical expression and management of the acute phase. Ital J Pediatr.

[3] Newburger JW, Takahashi M, Gerber MA (2004). Diagnosis, treatment, and long-term management of Kawasaki disease: a statement for health professionals from the Committee on Rheumatic Fever, Endocarditis, and Kawasaki Disease, Council on Cardiovascular Disease in the Young, American Heart Association. Pediatrics.

[4] Maggio MC, Corsello G, Prinzi E, Cimaz R (2016). Kawasaki disease in Sicily: clinical description and markers of disease severity. Ital J Pediatr.

[5] McCrindle BW, Rowley AH, Newburger JW (2017). Diagnosis, treatment, and long-term management of Kawasaki disease: a scientific statement for health professionals from the American Heart Association. Circulation.

[6] Marchesi A, Tarissi de Jacobis I, Rigante D (2018). Kawasaki disease: guidelines of Italian Society of Pediatrics, part II - treatment of resistant forms and cardiovascular complications, follow-up, lifestyle and prevention of cardiovascular risks. Ital J Pediatr.

[7] Saguil A, Fargo M, Grogan S (2015). Diagnosis and management of Kawasaki disease. Am Fam Physician.

[8] Wardle AJ, Connolly GM, Seager MJ, Tulloh RM (2017). Corticosteroids for the treatment of Kawasaki disease in children. Cochrane Database Syst Rev.

[9] Zhang M, Zheng Y, Li X, Yang S, Shi L, Li A, Liu Y (2022). Refractory Kawasaki disease: modified methylprednisolone regimen decreases coronary artery dilatation. Pediatr Res.

[10] JCS Joint Working Group (2010). Guidelines for diagnosis and management of cardiovascular sequelae in Kawasaki disease (JCS 2008)--digest version. Circ J.

[11] Fukazawa R, Kobayashi J, Ayusawa M (2020). JCS/JSCS 2020 guideline on diagnosis and management of cardiovascular sequelae in Kawasaki disease. Circ J.

[12] Wang L, Lin Y, Su YZ, Wang Y, Zhao D, Wu TJ (2004). Review and analysis of 283 cases of Kawasaki disease. (Article in Chinese). Zhonghua Er Ke Za Zhi.

[13] Maric LS, Knezovic I, Papic N, Mise B, Roglic S, Markovinovic L, Tesovic G (2015). Risk factors for coronary artery abnormalities in children with Kawasaki disease: a 10-year experience. Rheumatol Int.

[14] Saundankar J, Yim D, Itotoh B (2014). The epidemiology and clinical features of Kawasaki disease in Australia. Pediatrics.

[15] Seo YM, Kang HM, Lee SC (2018). Clinical implications in laboratory parameter values in acute Kawasaki disease for early diagnosis and proper treatment. Korean J Pediatr.

[16] Jeon SK, Kim G, Ko H, Byun JH, Lee HD (2019). Risk factors for the occurrence and persistence of coronary aneurysms in Kawasaki disease. Korean J Pediatr.

[17] Kim T, Choi W, Woo CW (2007). Predictive risk factors for coronary artery abnormalities in Kawasaki disease. Eur J Pediatr.

[18] Dominguez SR, Anderson MS, El-Adawy M, Glodé MP (2012). Preventing coronary artery abnormalities: a need for earlier diagnosis and treatment of Kawasaki disease. Pediatr Infect Dis J.

